# Accuracy of pedicle screw placement using neuronavigation based on intraoperative 3D rotational fluoroscopy in the thoracic and lumbar spine

**DOI:** 10.1007/s00402-022-04514-1

**Published:** 2022-07-06

**Authors:** Nora Conrads, Jan-Peter Grunz, Henner Huflage, Karsten Sebastian Luetkens, Philipp Feldle, Katharina Grunz, Stefan Köhler, Thomas Westermaier

**Affiliations:** 1 Department of Diagnostic and Interventional Radiology, University Hospital Würzburg, Oberdürrbacher Straße 6, 97080 Würzburg, Germany; 2 Department of Orthopedics and Trauma Surgery, Klinikum Würzburg Mitte – Standort Juliusspital, Juliuspromenade 19, 97070 Würzburg, Germany; 3 Department of Neurosurgery, University Hospital Würzburg, Josef-Schneider-Straße 11, 97080 Würzburg, Germany; 4 Die Neurochirurgie-Praxis, Eichhornstraße 28, 97070 Würzburg, Germany; 5 Department of Neurosurgery, Helios-Amper-Klinikum Dachau, Krankenhausstraße 15, 85221 Dachau, Germany

**Keywords:** Pedicle screws, Vertebral pedicles, Fluoroscopy, Neuronavigation, Spine

## Abstract

**Introduction:**

In spinal surgery, precise instrumentation is essential. This study aims to evaluate the accuracy of navigated, O-arm-controlled screw positioning in thoracic and lumbar spine instabilities.

**Materials and methods:**

Posterior instrumentation procedures between 2010 and 2015 were retrospectively analyzed. Pedicle screws were placed using 3D rotational fluoroscopy and neuronavigation. Accuracy of screw placement was assessed using a 6-grade scoring system. In addition, screw length was analyzed in relation to the vertebral body diameter. Intra- and postoperative revision rates were recorded.

**Results:**

Thoracic and lumbar spine surgery was performed in 285 patients. Of 1704 pedicle screws, 1621 (95.1%) showed excellent positioning in 3D rotational fluoroscopy imaging. The lateral rim of either pedicle or vertebral body was protruded in 25 (1.5%) and 28 screws (1.6%), while the midline of the vertebral body was crossed in 8 screws (0.5%). Furthermore, 11 screws each (0.6%) fulfilled the criteria of full lateral and medial displacement. The median relative screw length was 92.6%. Intraoperative revision resulted in excellent positioning in 58 of 71 screws. Follow-up surgery due to missed primary malposition had to be performed for two screws in the same patient. Postsurgical symptom relief was reported in 82.1% of patients, whereas neurological deterioration occurred in 8.9% of cases with neurological follow-up.

**Conclusions:**

Combination of neuronavigation and 3D rotational fluoroscopy control ensures excellent accuracy in pedicle screw positioning. As misplaced screws can be detected reliably and revised intraoperatively, repeated surgery for screw malposition is rarely required.

## Introduction

The lifetime prevalence of back pain in Germany is 85.5%, with men and women over the age of 50 being particularly affected [1]. Due to the increasing proportion of older people and the longer life expectancy in our population, it must be assumed that the medical and socioeconomic relevance of back pain and degenerative diseases of the spine will further increase [2]. In addition to debilitating degeneration, various underlying conditions such as trauma, inflammation, or neoplasms can be causative agents of spinal instability and require surgical treatment. The main goal of spinal surgery is to restore the spinal column’s weight-bearing capabilities and motion range of the spine in order to improve patients’ quality of life. To ensure this, a high degree of intraoperative precision is required for spinal instrumentation in patients with instabilities as screw misplacement can lead to neurological and vascular complications [3, 4]. Conventional X-ray diagnostics (2D radiographs or biplanar fluoroscopy) are widely regarded as the reference standard for intraoperative imaging in general, and for spinal instrumentation in particular [5]. However, while spinal alignment and vertebral body shape are sufficiently assessable in the far majority of cases, exact screw placement may occasionally be difficult to evaluate [6]. The question of whether modern navigation techniques can improve the precision of spinal instrumentation compared with conventional methods has not yet been clearly answered. Particularly, the application of 3D rotational fluoroscopy in combination with neuronavigation appears promising for intraoperative screw position analysis, as it provides multiplanar image information comparable to multidetector CT imaging.

The purpose of this retrospective study was to evaluate the accuracy of pedicle screw positioning in navigated, O-arm-controlled posterior instrumentation for the thoracic und lumbar spine.

## Material and methods

Retrospective data analysis was approved, and informed consent was waived by the local ethics committee. Information on patient history and surgical procedure was obtained from the clinical information system (SAP SE, Walldorf, Germany) and anonymized for further analysis. The evaluation of intraoperative imaging in terms of screw position and screw length was performed using an open-source DICOM viewer program (OsiriX Lite 8.0.1). For this study, we retrospectively analyzed data from patients who underwent dorsal spinal instrumentation with 3D fluoroscopic navigation (O-arm, Medtronic, Dublin, Ireland) at the local neurosurgical clinic between June 2010 to June 2015. Treatment indication was based on spinal instability due to degenerative, traumatic, inflammatory or tumor-related conditions. Inclusion criteria included surgical treatment via a dorsal approach (± additional fusion), surgery performed with an open or percutaneous technique and at least one rotational 3D fluoroscopy scan after dorsal instrumentation to evaluate the position of the screws. Patients who did not receive a rotational 3D scan were excluded from this study. Furthermore, screws that were not included in the field of view of the initial 3D fluoroscopy scan (*n* = 34) were also left out of the analysis.

The O-arm represents a 3D rotational fluoroscopy device designed for intraoperative application. In addition to the rotor, the gantry-based scanner architecture contains the X-ray tube (B100, Varian Medical Systems, Palo Alto, USA) opposite a large flat-panel detector (PaxScan 4030D, Varex, Palo Alto, USA). In 3D mode, the O-arm creates a series of projection images during a complete 360° rotation. Gantry rotation speed can be set to 30° per second in standard mode or 15° per second in high-definition mode with up to 400 or 750 images generated during a full 360° rotation. By integrating the navigation system (StealthStation S7, Medtronic) into the scanner setup, intraoperative imaging can be used directly for neuronavigation. This approach enables periprocedural display of entry points as well as identification of important neighboring structures.

**Fig. 1:**
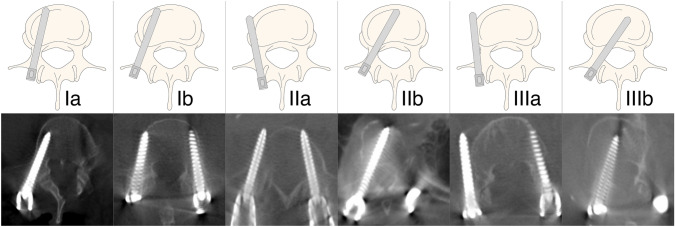
Visual representation of pedicle screw placement grading system. Schematic display and exemplary intraoperative 3D rotational fluoroscopy images of the classification system proposed by Zdichavsky et al. [7, 8]. Grading criteria are summarized in Table 1

Qualitative evaluation of screw positioning for the thoracic and lumbar spine was performed using the 6-grade scoring system described by Zdichavsky et al., in which grade Ia represents an excellent position, whereas grades IIIa and IIIb are supposed to be surgically revised [7, 8]. The classification system is based on the relative position of the inserted screw to the pedicle and vertebral body (Fig. 1, Table 1). In addition, the length ratio between screw and vertebral body diameter was calculated, with any relative screw length between 85 and 100% classified as good [9].

**Table 1:** Classification of pedicle screw placement

Graduation	Criteria
Ι a	≥ half of the PSD within the pedicle and ≥ half of the PSD within the vertebral body
Ι b	> half of the PSD laterally outside the pedicle and > half of the PSD within the vertebral body
ΙΙ a	≥ half of the PSD within the pedicle and > half of the PSD laterally outside the vertebral body
ΙΙ b	≥ half of the PSD within the pedicle and the tip of the PS crosses the midline of the vertebral body
III a	> half of the PSD laterally outside the pedicle and > half of the PSD laterally outside the vertebral body
III b	> half of the PSD medially outside the pedicle and the tip of the PS crosses the midline of the vertebral body

Graduation of screw positions in accordance with the classification system proposed by Zdichavsky et al. [7, 8]

***PSD*** pedicle screw diameter, ***PS*** pedicle screw

All data were transferred to a standard spreadsheet (Microsoft Excel for Mac, version 15.22, Redmond, USA) for further processing. Statistical analysis was performed using dedicated software (IBM SPSS Statistics, version 24.0.0.1 for Mac, Armonk, USA). Normal distribution was assessed with Kolmogorov–Smirnov tests. For normally distributed continuous variables, we report mean values and standard deviation, whereas absolute values and percentage distribution are displayed otherwise. Chi-square tests were applied to compare categorical data. To measure the effect size of the chi-square-test, Cramer-V was computed. Student’s *t*-tests were conducted to determine whether two normally distributed samples differ significantly. *P* values ≤ 0.05 were considered to indicate statistical significance.

## Results

Between June 2, 2010, and June 29, 2015, 285 patients (134 women, 47.0%) underwent 295 dorsal screw-rod instrumentations with 62 procedures performed on the thoracic and 233 on the lumbar spine. Lumbar vertebra 4 was stabilized most often (185 times) and thoracic vertebra 9 least often (14 times). Mean patient age at the time of surgery was 64.1 ± 12.6 years with almost 69% of patients over 60 years of age. Indication to perform surgical treatment was most frequently based on tumor-induced instability in the thoracic spine (46.8%) and degeneration-induced instability in the lumbar spine (76.8%). Table 2 summarizes the underlying conditions that led to surgical stabilization. Clinical follow-up was available in 238 patients (83.5%) with a mean time period of 14.3 ± 10.2 months.

**Table 2:** Underlying conditions for surgical indication

	Degeneration	Tumor	Trauma	Inflammation
Thoracic spine	11 (17.7%)	29 (46.8%)	11 (17.7%)	11 (17.7%)
Lumbar spine	179 (76.8%)	12 (5.2%)	21 (9.0%)	21 (9.0%)
Overall	190 (64.4%)	41 (13.9%)	32 (10.9%)	32 (10.9%)

Scale results are reported as absolute values (percentages)

Of 1704 included screws, 1621 (95.1%) showed an excellent position (Ia) in the initial intraoperative imaging. Of the remaining pedicle screws, 25 (1.5%) protruded the lateral rim of the pedicle (Ib), 28 (1.6%) protruded the lateral margin of the vertebral body (IIa), 8 (0.5%) crossed the vertebra’s midline (IIb). Furthermore, 11 screws each (0.6%) fulfilled the criteria of full lateral (IIIa) or medial displacement (IIIb). Median screw length was 92.6% of the maximum diameter of the vertebral body with 1224 screws (71.8%) displaying “good” length in the first scan.

**Fig. 2:**
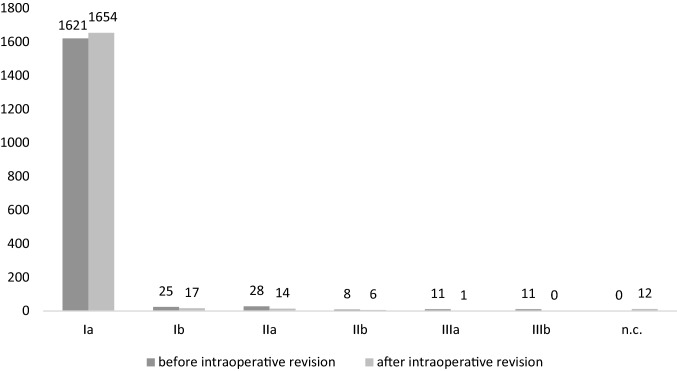
Screw position. Evaluation of screw positions according to the classification of Zdichavsky et al. [7, 8] before and after intraoperative revision of 71 screws

**Fig. 3:**
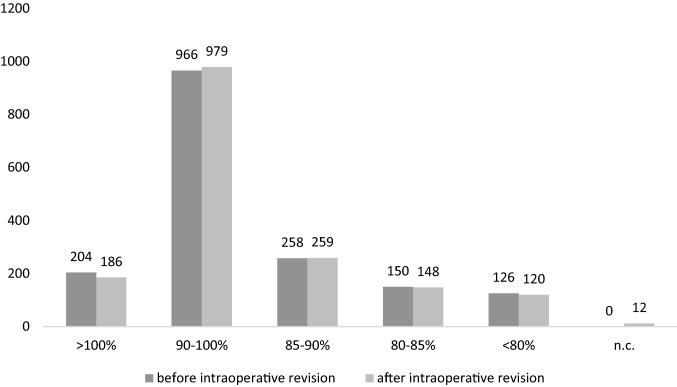
Relative screw length. Evaluation of relative screw length before and after intraoperative revision of 71 screws. Screw lengths between 100 and 85% are considered as “good”

After intraoperative revision, 58 of 71 screw positions were classified as Ia and one screw was classified as Ib. The other 12 revised screws were either not included in the field of view of the post-revision 3D scan or no further imaging was performed after intraoperative repositioning. Good relative length was ascertained in 1238 screws (72.7%). Screw position grading and relative length before and after intraoperative revision are summarized in Figs. 2 and 3, respectively. Repeated surgery was necessary in 11 patients (12 operations) with a total of 40 screws (2.3%) being repositioned. However, only two screws (0.1%) in one patient had to be revised due to primary malposition. The remaining screws had to be revised due to progressive loosening (20 screws [1.2%]), connection instability (15 screws [0.9%]) or inflammation-induced remodeling (3 screws [0.2%]).

At follow-up, 82.1% of patients declared total pain release or at least significant improvement of back and/or leg pain after surgery. No patient reported aggravating or new pain after surgery at the control examination. Neurological follow-up showed significant improvement or complete remission of symptoms in 70.8% of patients with neurological deficits. In contrast, 8.9% of patients with follow-up had new neurological symptoms that were not reported preoperatively.

## Discussion

In this study, the accuracy of pedicle screw placement in the thoracic and lumbar spine was investigated using a combined approach of neuronavigation and intraoperative 3D rotational fluoroscopy. High precision of implant positioning was achieved in all spinal sections. With a total of 1738 screws placed, intraoperative revision was performed for 78 screws, whereas repeated surgery due to a missed malposition was necessary in just one patient.

The classification system of Zdichavsky et al. represents a validated concept for determining the accuracy of pedicle screw placement in the thoracic and lumbar spine [7, 8]. Our results for placement accuracy are superior compared to the literature on screw positioning with conventional fluoroscopy-guidance [9, 10]. However, many earlier studies with similar designs use other forms of graduation, e.g., deviation from the ideal position in millimeter, dichotomous assessment of pedicle wall penetration [11] or screw placement < 50% or > 50% outside the pedicle [12]. Other studies only report accurate screw positioning when the thread is entirely intraosseous [13–17], or state misplacement solely in patients with postoperative neurological deficit or screws that require postoperative revision [18]. A meta-analysis by Gelalis et al. evaluated screw positioning with 3D fluoroscopy-guided neuronavigation, reporting accurate positioning for completely transpedicular screws in 81–92% of patients [19]. Assumedly, the inferior performance in individual studies within the meta-analysis compared to the present work may be attributed to substantially smaller patient samples with different sociodemographic characteristics. Besides, various definitions of screw misalignment yielded different shares of “correct” positioning. Different surgical indications, the experience of the surgeon, as well as the complexity of the surgery and height of the instrumented spinal segment also contributed to the heterogeneity of the results.

Revision surgery frequencies of up to 5.2% have been described in various studies on neuronavigated spinal surgery [20–22], which is considerably higher than in the present work. In the series presented here, only one of 285 patients required repeated procedures because of screw misplacement that was not detected intraoperatively. We assume that the far lower frequency of repetitive surgery can be attributed to the superior screw assessability provided by the O-arm-navigated approach, which is in line with the findings of Beck et al. [6].

Perdomo-Pantoja et al. showed in a recent meta-analysis on the accuracy of pedicle screw placement with different techniques that the highest accuracy results from CT navigation [23]. Nevertheless, it must be stated that high precision can also be achieved with free-hand or fluoroscopy-assisted screw insertion, even in patients with pronounced spinal deformities such as degenerative scoliosis [24]. Although Chan et al. demonstrated that screw breach rates are lower with CT navigation compared to free-hand methods, complication rates remained low with either technique [25].

Several limitations have to be addressed for this study. Since we performed a retrospective analysis, data quality regarding long-term outcome, neurological status and pain relief was inconsistent. Intraoperative revision rates of 4.2% were slightly higher than in comparable studies [6, 26]. However, we believe that this finding can be attributed to the inclusion of data from the introductory phase of the 3D fluoroscopy system. As degenerative diseases were predominantly responsible for spinal surgery in this study, decompression of spinal stenosis and/or cage insertion was frequently performed in addition to dorsal stabilization with a screw-rod system, hence affecting the clinical outcome. While 8.9% of patients with adequate follow-up reported new neurological symptoms, no association could be ascertained with misplaced screws that were revised intraoperatively.

## Conclusion

Combination of neuronavigation and 3D rotational fluoroscopy control ensures excellent accuracy in pedicle screw positioning. As misplaced screws can be detected reliably and revised intraoperatively, repeated surgery for screw malposition is rarely required.

## Data Availability

The datasets used and/or analyzed during the current study are available from the corresponding author on reasonable request.

## Abbreviations

CT: Computed tomography
PS: Pedicle screw
PSD: Pedicle screw diameter
